# Mathematical modelling the pathway of genomic instability in lung cancer

**DOI:** 10.1038/s41598-019-50500-w

**Published:** 2019-10-01

**Authors:** Lingling Li, Xinan Zhang, Tianhai Tian, Liuyong Pang

**Affiliations:** 1 School of Science, Xi’an Polytechnic University, Xi’an, 710048 P.R. China; 2 School of Mathematics and Statistics, Central China Normal University, Wuhan, 430079 P.R. China; 3 School of Mathematical Science, Monash University, Melbourne, Vic 3800 Australia; 4 School of Mathematics, Huanghuai University, Zhumadian, Henan P.R. China

**Keywords:** Lung cancer, Lung cancer

## Abstract

Genomic instability plays a significant role in lung cancer. Although substantial research has been conducted using both clinical and theoretical studies, it is still a hotly debated issue to whether genomic instability is necessary or whether genomic instability precedes oncogenes activation and tumor suppressor genes inactivation for lung cancer. In response to this issue, we come up with a mathematical model incorporating effects of genomic instability to investigate the genomic instability pathway of human lung cancer. The presented model are applied to match the incidence rate data of lung cancer from the Life Span Study cohort of the atomic bomb survivors in Nagasaki and Hiroshima and the Surveillance Epidemiology and End Results registry in the United States. Model results suggest that genomic instability is necessary in the tumorigenesis of lung cancer, and genomic instability has no significant impact on the net proliferation rate of cells by statistical criteria. By comparing the results of the LSS data to those of the SEER data, we conclude that the genomic instability pathway exhibits a sensitivity to radiation exposure, more intensive in male patients.

## Introduction

Genomic instability (GI) is a important hallmark of almost all human cancers^1^, which is a very active area of research in cancer biology. There are two main types of GI in human cancer^2^. One is mircrosatellite instability (MIN) which is featured by the amplification or deletion of oligonucleotide number in microsatellite sequences^3–5^. The other is chromosomal instability (CIN) that involves the acquisition or loss of large parts of chromosomes or entire chromosomes during the period between cell divisions. However, MIN is rarely spotted in cancers but colon cancer. CIN is widespread among solid tumors^6,7^. As is known to all, the tumorigenesis is caused by the accumulated alterations in oncogenes genes, tumor suppressor genes and genes controlling genomic stability^8^. Alterations in tumor suppressor genes and oncogenes genes affect the clonal expansion of cells, while alterations in genes controlling genomic stability accelerate the mutational processes such as chromosome recombination, loss or gain of large parts of chromosomes or entire chromosomes, gene amplification and so on^2,8^. Thus, GI drives the development of cancers by increasing the spontaneous mutation rate.

Biologically-based mechanistic model is a useful tool to study the initiation and progression of cancer. Nowell presented a carcinogenesis model based on clonal selection and GI in 1976^9^. Later, Tomlinson and Bodmer proposed that tumor processes is mainly result from selection of clonal expansion of mutated cells^10^. However, Loeb introduced the concept of mutator phenotype, which suggests that mutation in a stability gene is a necessary event in carcinogenesis^11,12^. In addition, a large amount of experimental and theoretical studies has been conducted to analyze the mechanism of GI in the development of cancer^13–17^. Nowak *et al*.^13–15^ presented a mathematical model with CIN to explore the dynamics of CIN in the progression of colon cancer by means of the modification to a two-stage clonal expansion model given by Moolgavkar *et al*.^18^. Based on this mathematical framework, the model with multiple genetic pathway was set up to study cancer^19–22^. Z*ö*llner *et al*.^23^ applied the model incorporated GI to match the mortality data of lung cancer in the Mayak-workers cohort and study the effect of plutonium on the risk of lung cancer.

Lung cancer is the leading cause of cancer mortality all over the world^24,25^, and the second leading cause of cancer mortality in the Life Span Study (LSS) cohort of the atomic bomb survivors in Nagasaki and Hiroshima^26^. Cigarette smoking and radiation are two main factors to increase the risks of lung cancer^26,27^. Evidence indicates that GI plays a curial role in the initiation and progression of lung cancer^28–31^. Nearly all lung cancer patients exhibit GI which is resulted in an abnormal number of chromosomes known as aneuploidy^32,33^. CIN is an important DNA alteration process for lung cancer, which is a significant poor prognostic factor^34,35^. In addition, cancer risk may be attributed to GI, which plays a vital role in carcinogenesis and in clinical practice. Nevertheless, the mechanisms of GI in the development lung cancer are still unclear. Therefore, understanding the role of GI is very imperative to guide therapeutic interventions for lung cancer.

It is very important to determine whether GI is an early event or a late event in lung cancer development, which is a long-standing debate in cancer genetics. In this article, we describe a model with GI to analyze the pathways of GI in the initiation and progression of lung cancer. Using the age-specifical incidence rate data of lung cancer from the LSS cohort in Nagasaki and Hiroshima during 1958–1987 and the Surveillance Epidemiology and End Results (SEER) registry in the United States during 1993–2012 as the study system, the model with GI is applied to match these data. We firstly discuss the impact of GI on the net reproduction rate of cells and then analyze the issues that whether GI is necessary for the process from normal stem cells into a malignant cell in the lung tissue, and whether GI precedes oncogenes activation and tumor suppressor genes inactivation for lung cancer.

## Methods

### The LSS data

The LSS cohort of the atomic bomb survivors in Nagasaki and Hiroshima is a primary data source for analyzing the impact of radiation on cancer risk, which involves 120,321 persons. Lung cancer occupies almost 10% of all cancers in the LSS cohort. The LSS data are stratified by region (Nagasaki and Hiroshima), sex (male and female), attained age (ages 0–85+), age at exposure (ages 0–60+), calendar time (1958–1987), colon dose (radiation dose detected in the colon), and other factors^26^. These data can be obtained at the Radiation Effects Research Foundation (RERF) website (http://www.rerf.jp). The detailed description of the data can be found in the refs^26,36^. There is no significant difference in patients between Nagasaki and Hiroshima^36,37^. Therefore, our analyses are based on adjusted person-years and incidence cases of lung cancer grouped by sex and attained age for calendar years 1958–1987 in Nagasaki and Hiroshima (see Table 1). In our study, the lung cancer data for persons between the age of 0 and 29 years are ignored since they are equal to zero.

**Table 1:** The age-specific lung cancer incidence data of male and female from the LSS cohort of the atomic bomb survivors in Hiroshima and Nagasaki for the year 1958–1987.

Age	Cases		Person years	
	Male	Female	Male	Female
30–34	1	0	686002	854695
35–39	1	7	657211	1019250
40–44	10	3	644822	1129502
45–49	13	12	560137	1028308
50–54	19	31	584624	1189838
55–59	37	33	461360	1058327
60–64	70	49	493031	947685
65–69	122	62	489571	813978
70–74	115	72	323201	614319
75–79	69	47	188111	372679

### The SEER data

Lung cancer incidence data were obtained from the SEER registry in the United States for the years 1975–2012 (www.seer.cancer.gov)^38^. The reported lung cancer incidence data were grouped by sex (male and female), age (0–85+), and calendar year (1975–2012) in the eighteen SEER geographic areas^39^. The SEER population files (data from U.S. Census Bureau) provided the population bases, which was stratified by gender (male and female), calender year (1975–2012) and age (ages 0–85+). Our analyses employed lung cancer incidence data of all races in males and females for the years 1993–2012. We considered the data for persons aged 30–79 since the lung cancer incidence rates are closed to zero for the age group 0–29. The detailed data are displayed in Table 2.

**Table 2:** The age-specific lung cancer incidence data of male and female from SEER registry for the year 1993–2012.

Age	Cases		Person years	
	Male	Female	Male	Female
30–34	517	545	45116274	43026403
35–39	1521	1649	45911554	44503264
40–44	4813	4741	46042363	45269976
45–49	11742	11041	43781154	43664229
50–54	23596	19595	38983073	39518370
55–59	37866	29581	32164300	33232561
60–64	52933	41006	25341059	26903037
65–69	66214	53061	19538332	21835643
70–74	70736	58317	15426031	18524069
75–79	65406	56679	12003384	16000285

### Mathematical model

**Figure 1:**
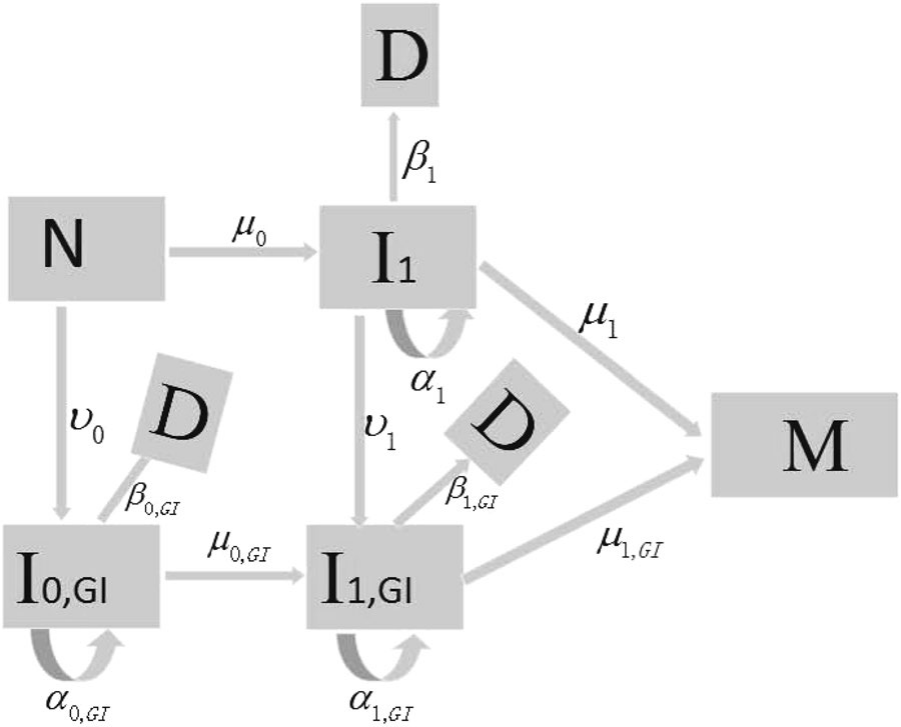
The schematic representation of GI model for carcinogenesis. *N* denotes the normal cell; *I*_1_ the compartment of intermediate cell without GI; *I*_*i*_,_*GI*_ (*i* = 0, 1) the compartment of intermediate cell with GI; D the dead or differentiated cell; M the malignant cell. *μ*_*i*_(*t*), *μ*_*i*_,_*GI*_(*t*) are the mutation rates per cell per year at time *t*, respectively. *α*_1_(*t*), *β*_1_(*t*), *α*_*i*_,_*GI*_(*t*) and *β*_*i*_,_*GI*_(*t*) are the growth rate and death rate per cell per year at time *t*, respectively.

CIN and the loss of heterozygosity play important roles in the progression of lung cancer^40^. The predominant form of GI in lung tumors is CIN. Only a single mutational ‘hit’ is required to produce the CIN phenotype^2^. Thus, we assume that cell mutations occur along two different pathways in lung cancer development. The model is illustrated schematically in Fig. 1. The first (upper) path does not generate GI, whereas the second (below) path is activated via transition rates *υ*_*i*_ (*i* = 0, 1), corresponding to the alteration of genes in maintaining genomic integrity or stability. CIN accelerates the rate of inactivating tumour suppressor genes and activating oncogenes. Therefore mutation rates in the cells with GI are higher than those of the cells without GI, *μ*_*i*_,_*GI*_ ≫ *μ*_*i*_^8,23^. When all transition rates *υ*_*i*_ (*i* = 0, 1) equal zero, the model corresponds to the two-stage model. Our model assumes that malignant tumor occurs with probability one if a malignant cell is generated. Therefore, the mutation rates *μ*_1_ and *μ*_1_,_*GI*_ are to be regarded as net or effective mutation rates, which means that various defense mechanisms are taken into account in our model such as immune surveillance. As described in the ref. ^26^, we suppose that there are *N* normal stem cells in the lung tissue; any normal cell mutates to premalignant cells in compartment *I*_1_ at a rate of *μ*_0_; and a type of cells with GI at a rate of *υ*_0_. After that, any mutated cell in compartment *I*_1_ (*I*_1_,_*GI*_) forms two equivalent daughter cells at a rate *α*_1_ (*α*_1_,_*GI*_); and an equivalent daughter cell and an mutated cell in compartment *M* at a rate *μ*_1_ (*μ*_1_,_*GI*_); and die or differentiation at a rate *β*_1_ (*β*_1_,_*GI*_). Each mutated cell in compartment *I*_1_ can also turn into an daughter cell and an mutated cell with GI at a rate *υ*_1_.

In the model, we let *X*_1_(*t*) to denote the number of mutated cells without GI (compartment *I*_1_) at time *t*, *Y*_*i*_(*t*) (*i* = 0, 1) the number of mutated cells with GI (compartment *I*_*i*_, _*GI*_) at time *t*, and *Z*(*t*) the number of fully malignant cells (compartment M) at time *t*. We define the probability generating functions as follows:

1 $$\begin{array}{rcl}\psi ({x}_{1},{y}_{0},{y}_{1},z;t) & = & \sum _{{i}_{1},{j}_{0},{j}_{1},k}\,p\{{X}_{1}(t)={i}_{1},{Y}_{0}(t)={j}_{0},{Y}_{1}(t)={j}_{1},Z(t)=k|\\  &  & {X}_{1}(0)=0,{Y}_{0}(0)=0,{Y}_{1}(0)=0,Z(0)=0\}{x}_{1}^{{i}_{1}}{y}_{0}^{{j}_{0}}{y}_{1}^{{j}_{1}}{z}^{k},\end{array}$$

2 $$\begin{array}{rcl}\phi ({x}_{1},{y}_{1},z;t) & = & \sum _{{i}_{1},{j}_{1},k}\,p\{{X}_{1}(t)={i}_{1},{Y}_{1}(t)={j}_{1},Z(t)=k|{X}_{1}(0)=1,{Y}_{1}(0)=0,\\  &  & Z(0)=0\}{x}_{1}^{{i}_{1}}{y}_{1}^{{j}_{1}}{z}^{k},\end{array}$$

3 $$\begin{array}{rcl}{\varphi }_{0}({y}_{0},{y}_{1},z;t) & = & \sum _{{j}_{0},{j}_{1},k}\,p\{{Y}_{0}(t)={j}_{0},{Y}_{1}(t)={j}_{1},Z(t)=k|{Y}_{0}(0)=1,{Y}_{1}(0)=0,\\  &  & Z(0)=0\}{y}_{0}^{{j}_{0}}{y}_{1}^{{j}_{1}}{z}^{k},\end{array}$$

and

4 $${\varphi }_{1}({y}_{1},z;t)=\sum _{{j}_{1},k}\,p\{{Y}_{1}(t)={j}_{1},Z(t)=k|{Y}_{1}(0)=1,Z(0)=0\}{y}_{1}^{{j}_{1}}{z}^{k},$$

where *x*_1_, *y*_0_, *y*_1_ and *z* are the arguments for these probability generating functions.

These probability generating functions satisfy the Kolmogorov backward equations as follows^41–43^:

5 $$\{\begin{array}{rcl}\frac{d\phi }{dt}(t) & = & -[{\alpha }_{1}+{\beta }_{1}+{\mu }_{1}+{\upsilon }_{1}]\,\phi (t)+{\alpha }_{1}{\phi }^{2}(t)+{\upsilon }_{1}\phi (t)\,{\varphi }_{1}(t)+{\mu }_{1}\phi (t)\,z+{\beta }_{1}\\ \frac{d{\varphi }_{0}}{dt}(t) & = & -[{\alpha }_{0,GI}+{\beta }_{0,GI}+{\mu }_{0,GI}]\,{\varphi }_{0}(t)+{\alpha }_{0,GI}\,{\varphi }_{0}^{2}(t)+{\mu }_{0,GI}\,{\varphi }_{0}(t)\,{\varphi }_{1}(t)+{\beta }_{0,GI}\\ \frac{d{\varphi }_{1}}{dt}(t) & = & -[{\alpha }_{1,GI}+{\beta }_{1,GI}+{\mu }_{1,GI}]\,{\varphi }_{1}(t)+{\alpha }_{1,GI}\,{\varphi }_{1}^{2}(t)+{\mu }_{1,GI}\,{\varphi }_{1}(t)z+{\beta }_{1,GI}\end{array},$$

and

6 $$\frac{d\psi }{dt}(t)={\mu }_{0}N\psi (t)\,[\phi (t)-1]+{\upsilon }_{0}N\psi (t)\,[{\varphi }_{0}(t)-1],$$

where we have dropped the arguments *x*_1_, *y*_0_, *y*_1_ and *z* in *ψ*, *φ*, *ϕ*_0_ and *ϕ*_1_ for simplicity. The detailed derivation for above equations can be seen in Appendix.

Here, the hazard function, *h*(*t*), signifies the cancer incidence rate at time *t*. It can be written as^18,39,41^

7 $$h(t)=-\frac{d\psi (1,1,1,0;t)}{dt}/\psi (1,1,1,0;t).$$

It follows from Eq. (6) that

8 $$h(t)=-\,{\mu }_{0}N\,[\phi (1,1,0;t)-1]-{\upsilon }_{0}N\,[{\varphi }_{0}(1,1,0;t)-1].$$

Hence, the solutions of the hazard function, *h*(*t*), can be obtained by solving *φ*(1, 1, 0; *t*) and *ϕ*_0_(1, 1, 0; *t*). By above probability generating functions, we have $\phi (1,1,0;0)=1$ and ${\varphi }_{i}(1,1,0;0)=1$ (*i* = 0, 1). Thus, the solutions of $\phi (1,1,0;t)$ and ${\varphi }_{i}(1,1,0;t)$ can be obtained by Eq. (5) with the initial conditions $\phi (1,1,0;0)=1$ and ${\varphi }_{i}(1,1,0;0)=1$.

For the two-stage model (*υ*_0_ = *υ*_1_ = 0), however, there is a closed-form solution of hazard function given by,

9 $$h(t)=\frac{{\mu }_{0}N}{{\alpha }_{1}}(\frac{pq({e}^{-qt}-{e}^{-pt})}{q{e}^{-pt}-p{e}^{qt}}),$$

where $$p,{q}\,:\,=\frac{1}{2}(\,-\,({\alpha }_{1}-{\beta }_{1}-{\mu }_{1})\mp \sqrt{{({\alpha }_{1}-{\beta }_{1}-{\mu }_{1})}^{2}+4{\alpha }_{1}{\mu }_{1}})$$.

### Parameter estimation

Given set of lung cancer cases {*o*_*a*_} with corresponding adjusted person-years {*n*_*a*_}, we derive the likelihood function for the model in the following way^39,44^. The number of lung cancer incidence cases is assumed to be a Poisson distribution with mean *λ*_*a*_ = *n*_*a*_*h*(*a*), where *h*(*a*) is the hazard function with parameter set Θ = (*N*, *α*_1_, *β*_1_, *α*_*i*_,_*GI*_, *β*_*i*_,_*GI*_, *μ*_*i*_, *μ*_*i*_,_*GI*_, *υ*_*i*_)^39^. Lung cancer cases are supposed to be independent, and then the likelihood function for entire data set of lung cancer cases {*o*_*a*_} can be written as

10 $$L(\Theta )={\Pi }_{a}\frac{exp\,\{\,-\,{\lambda }_{a}\}{\lambda }_{a}^{{o}_{a}}}{{o}_{a}!}.$$

The negative log likelihood (NLL) function can be written as

11 $$NLL(\Theta )=-\,\sum _{a}\,(\,-\,{\lambda }_{a}+{o}_{a}log\,{\lambda }_{a}-log{o}_{a}!).$$

The NLL of the model is minimized by using the optimization routine fminsearch in MATLAB. The optimal parameters are obtained by minimizing the deviance, *Dev* = 2 *NLL*. We use the AIC to measure the goodness of model fit, which can availably avoid overfitting of models^45^. The AIC is equal to 2(*NLL* + *n*) where *n* denotes the number of model parameters. Hence, a smaller AIC represents a better fitting result.

### Analysis of genetic instability

GI has long been hypothesized to be a critical feature of cancer. However, the mechanism of GI is still not completely clear for lung cancer. In this article, we mainly pay attention to the questions that whether GI is necessary for lung cancer, and whether GI precedes oncogenes activation and tumor suppressor genes inactivation if GI occurs in the development of lung cancer. Hence, we consider the model with GI. For the model with GI, the following two cases are discussed: namely (1) GI is identified in the earliest stage of tumorigenesis (*υ*_0_ ≠ 0); (2) GI occurs after inactivation of tumor suppressor genes or activation of oncogenes (*υ*_0_ = 0). The LSS data and the SEER data are applied to analyze these issues. For the LSS data, the lung cancer cases are strongly correlated to radiation exposure, which can be used to examine the effect of radiation exposure on GI by comparing with the fitting results of the SEER data.

## Results

Results from the two-stage model are shortly presented. The changes of clonal expansion in premalignant cells due to GI are investigated. Furthermore, the GI model with different pathway is discussed in detail.

### The two-stage model

For the two-stage model (*υ*_0_ = *υ*_1_ = 0), we derive the closed-form solution of the hazard function (9). This model has three identifiable parameter combinations, $\frac{{\mu }_{0}N}{{\alpha }_{1}}$, *p* and *q*. We can obtain the following formulas by the expressions of *p* and *q*,

12 $$\{\begin{array}{rcl}p+q & = & -({\alpha }_{1}-{\beta }_{1}-{\mu }_{1})\\ pq & = & -{\alpha }_{1}{\mu }_{1}\end{array},$$

Therefore, the *α*_1_ − *β*_1_ − *μ*_1_, *α*_1_*μ*_1_ and $\frac{{\mu }_{0}N}{{\alpha }_{1}}$ can be determined from the data. Here, we assume that the latent time from a malignant cell to clinical detection is 5 years^23,26^. By fitting the LSS data and the SEER data, we obtain the values of these parameter combinations, which is displayed in Table 3. By these values of parameters in Table 3, we obtain that the net proliferation of premalignant cells in the SEER data is slightly larger than that in the LSS data, and the transformation rate from the premalignant cells to malignant cell for LSS data is larger than that for SEER data. If we fix the values of *α*_1_ and *N*, the other parameter values can be determined. Similarly, we set the values of cell mutation rates to be equal, *μ*_0_ = *μ*_1_, and know the number of stem cells in the lung tissue, *N*, this identifiability problem can also be solved. The hazard function mainly rely on the net growth rate of cells, *α*_1_ − *β*_1_, and is insensitive to a broad range of assumed value for *α*_1_. Thus we set the growth rate to *α*_1_ = 12 per year^39,46^. If we accept the estimate of *N* provided by Hazelton *et al*.^47^, about 10^7^, the other parameters in the two-stage model can be identified.

**Table 3:** The estimated values of parameter combinations in the two-stage model by fitting the SEER data and the LSS data respectively.

Parameters	The LSS data		The SEER data	
	Male patients	Female patients	Male patients	Female patients
$\frac{{\mu }_{0}N}{{\alpha }_{1}}$	0.0031	0.0015	0.0356	0.0253
*p*	−0.1584	−0.1226	−0.1718	−0.1610
*q*	0.9622 × 10^−5^	5.3857 × 10^−5^	0.6232 × 10^−5^	1.2465 × 10^−5^

### The model with GI

For the model with GI, there is no analytical solution and more non-identifiability parameters. Generically, the widely used approach to deal with this non-identifiability problem is to set the background mutation rates equal to each other and assume a reasonable value for some parameters or use a new set of parameters^48,49^. Therefore, we give the following limitations:

- the mutation rates in cells are assumed as *μ*_0_ = *μ*_1_ and *μ*_0_,_*GI*_ = *μ*_1_,_*GI*_, which is reasonable by fitting the data.
- the birth rate and death (differentiation) rate in the compartment *I*_0_,_*GI*_ are the same, namely *α*_0_, _*GI*_ = *β*_0_,_*GI*_, since cells without mutation of oncogene or tumor suppressor gene have not growth advantage.
- the instability transition rates are equal, namely *υ*_0_ = *υ*_1_, since they belong to the inactivation of the same type genomic-integrity gene.

In the GI model, the transition rates to achieve GI mainly depend on the number of dominant CIN genes, a reasonable range of the rate is from 10^−7^ to 10^−5^ ^50^. Here, we discuss the effect of GI on clonal expansion of cells. The following three scenarios are considered:

(i) GI decreases the rate of the cell clonal expansion, that is *γ*_1_ = *α*_1_ − *β*_1_ larger than *γ*_1_,_*GI*_ = *α*_1_,_*GI*_ − *β*_1_,_*GI*_.
(ii) GI has no effect on clonal expansion of cells, that is *γ*_1_ = *α*_1_ − *β*_1_ equals *γ*_1_,_*GI*_ = *α*_1_,_*GI*_ − *β*_1_,_*GI*_.
(iii) GI increases the rate of the cell clonal expansion, that is *γ*_1_ = *α*_1_ − *β*_1_ smaller than *γ*_1_,_*GI*_ = *α*_1_,_*GI*_ − *β*_1_,_*GI*_.

The comparison of the models with these three scenarios is shown in Table 4. By Table 4, we find that the values of AIC for the scenario, *γ*_1_ = *γ*_1_,_*GI*_ are the smallest apart from male patients in the SEER data. However, the values of AIC for the three scenarios do not change very much for male patients in the SEER data. This suggests that GI has no significant effect on clonal expansion of cells in the lung cancer progression. Hence, we choose the GI model with *γ*_1_ = *γ*_1_,_*GI*_.

**Table 4:** Comparison of the GI models with the three different hypotheses for the effect of GI on clonal expansion of cells in the development of lung cancer.

Model	No. Parameter	The LSS data				The SEER data			
		Male patients		Female patients		Male patients		Female patients	
		Deviance	AIC	Deviance	AIC	Deviance	AIC	Deviance	AIC
*γ*_1_ > *γ*_1_,_*GI*_	5	52.684	62.684	65.677	75.677	1094.5	1104.5	1169.3	1179.3
*γ*_1_ = *γ*_1_,_*GI*_	4	52.615	60.615	61.272	69.272	1065.5	1073.5	1132.6	1140.6
*γ*_1_ < *γ*_1_,_*GI*_	5	52.399	62.399	61.409	71.409	1032.6	1042.6	1132.4	1142.4

### Model comparison

**Figure 2:**
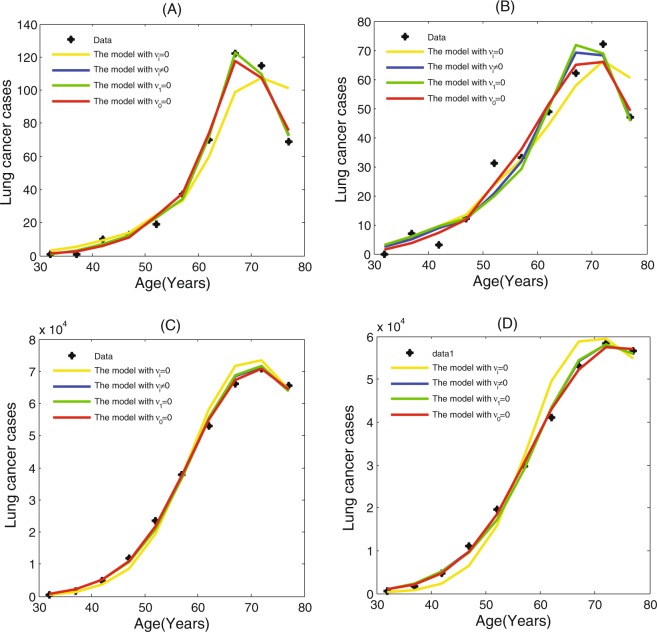
The age-specific lung cancer cases data for male patients and female patients from the LSS cohort for the year 1958–1987 and the SEER registry for the year 1993–2012, and cases predicted by the models. (**A**) Prediction for male patients in the LSS data. (**B**) Prediction for female patients in the LSS data. (**C**) Prediction for male patients in the SEER data. (**D**) Prediction for female patients in the SEER data.

A comparison of the GI model for different pathway is presented in Table 5. The results indicate that the model with GI is a significant improvement on model accuracy compared to the model without GI for the SEER data. It has been shown that the model with GI has better goodness-of-fit than the model without GI for the SEER data. For the LSS data, the fitting result of the model with GI is better than that of the model without GI for male patients, and the optimal model is the model with *υ*_1_ = 0 for female patients. Hence, we can obtain that GI is needed in the development of lung cancer. By the values of AIC in Table 5, we find that there are significant difference in the fitting results for the SEER data and those for the LSS data. For the SEER data, the AIC value of the model with *υ*_1_ = 0 is far less than the other models for male patients and female patients. It turn out that GI occurs after oncogenes activation or tumor suppressor genes inactivation in the lung cancer development. For the LSS data, however, the model with *υ*_0_ = *υ*_1_ ≠ 0 and the one with *υ*_1_ = 0 have the smaller AIC than the one with *υ*_0_ = 0 for male patients. The preferred model is the model with *υ*_1_ = 0 for male patients. For female patients in the LSS data, however, the GI model with *υ*_1_ = 0 has the smaller AIC than the other models, and the AIC values of the model with different pathway do not get too much of a bump. This difference between the fitting results for the SEER data and those for the LSS data is due to radiation exposure, since the LSS data is strongly associated with radiation exposure. Therefore, we infer that radiation exposure can induce the mutation of genes in maintaining genomic integrity or stability for lung cancer, especially for male lung cancer patients. The fitting results of the model with different pathway are displayed in Fig. 2, which indicates that the model with GI is superior to the model without GI for the fitting of the lung cancer incidence data.

**Table 5:** Comparison of the GI model with the different pathway.

Model	No. Parameter	The LSS data				The SEER data			
		Male patients		Female patients		Male patients		Female patients	
		Deviance	AIC	Deviance	AIC	Deviance	AIC	Deviance	AIC
Without GI (*ϑ*_*i*_ = 0)	2	77.415	81.415	64.118	68.118	3217.5	3221.5	8648.3	8652.3
Early GI (*ϑ*_*i*_ ≠ 0)	4	52.615	60.615	61.272	69.272	1065.5	1073.5	1132.6	1140.6
Early GI (*ϑ*_1_ = 0)	4	52.560	60.560	65.199	73.199	1110.3	1118.3	1196.5	1204.5
Late GI (*ϑ*_0_ = 0)	4	55.167	63.167	57.242	65.242	691.5	699.5	600.1	608.1

*i* = 0, 1.

## Discussion

whether GI is necessary for lung cancer or what stage of lung cancer development it arises remain hotly debated. In this paper, we have proposed a model with GI to investigate the issue. The LSS data closely related with radiation exposure^51^ are used to study the pathway of GI in the development of lung cancer. To eliminate the effect of radiation on GI, the SEER data are also applied to address this issue. Our results suggest that GI is highly significant for lung cancer in the SEER data. By the comparing the inference results from the model with different pathway, we obtain that GI is a late event in lung cancer development for the SEER data, while for male patients in the LSS data GI is most likely to occur before oncogenes activation and tumor suppressor genes inactivation. In addition, for female patients in the LSS data, the model with *υ*_1_ = 0 has no more significant improvement than the other models. Therefore, we conclude that the pathway of GI is sensitive to radiation in lung cancer development, and the sensitivity is more intensive for male patients.

CIN is the most common type of GI in most cancers, which leads to the high gene mutation rate by chromosome number and structure alterations over time in mutated cells with CIN compared with the cells without GI^5^. CIN is the dominating form of GI in lung tumors. Thus the mutation rate of GI in the model typically relys on the number of genes controlling chromosome stability in the genome. Our work mainly pays attention to the relationship between GI and lung cancer and the question that whether GI precedes oncogenes activation and tumor suppressor genes inactivation. Two different data cohorts are chosen to address these issues. The LSS data is a main data source used for analyzing the cancer risks from radiation exposure^26,52,53^, and the SEER data is widely used to study the development of cancer^26,54–56^. By the fitting of these data, we obtain that GI has no significant influence on the clonal expansion of cells, and the model with GI offers an improved description of the data. In addition, radiation exposure can affect the mechanism of GI in the progression of lung cancer. These conclusions will not only contribute to understanding the mechanisms of lung tumorigenesis, but also provide constructive suggestions for the prediction, diagnosis and treatment of lung cancer.

Although a number of works have been designed to study lung cancer, there is limited work to discuss the mechanism of GI in lung cancer by using the detailed mathematical framework. The major contribution of our work is to discuss the effect of GI on the net reproduction rate of cells and the pathway of GI in lung cancer development by the GI model. However, our proposed model can not account for the specific genes involving the GI pathway in the initiation and progression of lung cancer, and the results are obtained only by considering the incidence rate of lung cancer. More detailed biological and medical data are needed to further support our results. Besides, the GI pathway in different types of lung cancer may be different, which requires some additional information to analyze it. Recently, research demonstrated that radiation exposure is mainly affecting the pathway with transmembrane receptor–mutant for lung adenocarcinoma by connecting molecular biology with epidemiology^53^. Hence, the model incorporated growing knowledge on carcinogenesis processes is required to further study the mechanism of GI in lung cancer.

## Supplementary information

Appendix
