# Supplementary material for: Mathematical modelling the pathway of genomic instability in lung cancer
Source: Sci Rep. 2019 Oct 1;9:14136. doi: 10.1038/s41598-019-50500-w (PMC6773729; doi:10.1038/s41598-019-50500-w)
Supplement: Supplementary file 1 — Appendix [file 41598_2019_50500_MOESM1_ESM.pdf]

Mathematical modelling the pathway of genomic instability in lung cancer

Lingling Li[1]*, Tianhai Tian[2], Xinan Zhang[3] and Liuyong Pang[4]

[1]School of Science, Xi'an Polytechnic University, Xi'an, 710048, P.R.China

[2]School of Mathematical Science Monash University Melbourne Vic 3800, Australia

[3]School of Mathematics and Statistics Central China Normal University, Wuhan 430079, P.R.China

[4]School of Mathematics, Huanghuai University, Zhumadian, Henan, PR China

Appendix

In this appendix, the differential equation for φ is given, the derivations of other probability generating functions can be obtained similarly. For the multistage cancer models, the Kolmogorov backward equations provide a useful tool for deriving the probability of first entry into the malignant state, and the associated hazard function.

We consider the $\varphi(t+\Delta)$, where Δ is very small increment in time. By the Chapman-Kolmogorov equations, the $\varphi(t+\Delta)$ can be written as

$$\begin{aligned}\varphi(t+\Delta) \; &= \sum_{i_1,j_1,k}\sum_{m_1,n_1,w} p\Big\{X_1(t+\Delta)=i_1, Y_1(t+\Delta)=j_1, Z(t+\Delta)=k|X_1(\Delta)=m_1,\\ &Y_1(\Delta)=n_1, Z(\Delta)=w\Big\}p\Big\{X_1(\Delta)=m_1, Y_1(\Delta)=n_1, Z(\Delta)=w|X_1(0)=1,\\ &Y_1(0)=0, Z(0)=0\Big\}x_1^{i_1}y_1^{j_1}z^l.\end{aligned}$$

Because the number of events that can occur in a sufficiently small amount of time is at most one, the probabilities of the events that can occur in $[0,\Delta]$ are approximately $\Delta\alpha_1$ for the birth of a state I_1 cell, $\Delta\beta_1$ for the death or differentiation of a state I_1 cell, $\Delta\mu_1$ for the transformation from state I_1 to M, $\Delta\upsilon_1$ for the transformation from state I_1 to $I_{1,GI}$, and $1-\Delta\alpha_1-\Delta\beta_1-\Delta\mu_1-\Delta\upsilon_1$ for no change in the

*Corresponding author email:linglinglimath@163.com

system, respectively. Thus, it can be derived that

$$\begin{aligned}\varphi(t+\Delta) = \sum_{i_1,j_1,k} \Big\{ & \Delta\alpha_1 p\Big\{X_1(t+\Delta)=i_1, Y_1(t+\Delta)=j_1, Z(t+\Delta)=k|X_1(\Delta)=2, \\ & Y_1(\Delta)=0, Z(\Delta)=0\Big\} + \Delta\beta_1 p\Big\{X_1(t+\Delta)=i_1, Y_1(t+\Delta)=j_1,\ Z(t+\Delta) \\ & =k|X_1(\Delta)=0, Y_1(\Delta)=0, Z(\Delta)=0\Big\} + \Delta\mu_1 p\Big\{X_1(t+\Delta)=i_1, Y_1(t+\Delta) \\ & =j_1, Z(t+\Delta)=k|X_1(\Delta)=1, Y_1(\Delta)=0, Z(\Delta)=1\Big\} + \Delta\upsilon_1\ p\Big\{X_1(t+\Delta) \\ & =i_1, Y_1(t+\Delta)=j_1, Z(t+\Delta)=k|X_1(\Delta)=1, Y_1(\Delta)=1, Z(\Delta)=0\Big\} \\ & +(1-\Delta\alpha_1-\Delta\beta_1-\Delta\mu_1\Delta\upsilon_1)p\Big\{X_1(t+\Delta)=i_1, Y_1(t+\Delta)=j_1, \\ & Z(t+\Delta)=k|X_1(\Delta)=1, Y_1(\Delta)=0, Z(\Delta)=0\Big\}\Big\} x_1^{i_1} y_1^{j_1} z^k.\end{aligned}$$

The full probability generating function can be expressed as the product of individual probability generating functions with initial conditions of only one cell in one state raised to the power of the correct number of initial cells.[1,2] The situation considered is a time-homogeneous process (the parameters of the model are constant). By the definition for $\varphi(t)$ and Markov property, it is easy shown that

$$\begin{aligned}\varphi(t+\Delta) &= \Delta\alpha_1\varphi^2(t) + \Delta\beta_1 + \Delta\mu_1\varphi(t)z + \Delta\upsilon_1(t)\phi_1(t) \\ &\quad +(1-\Delta\alpha_1-\Delta\beta_1-\Delta\mu_1-\Delta\upsilon_1)\varphi(t),\end{aligned}$$

consequently,

$$\frac{d\varphi}{dt}(t) = -(\alpha_1+\beta_1+\mu_1+\upsilon_1)\varphi(t) + \alpha_1\varphi^2(t) + \mu_1\varphi(t)z + \upsilon_1\varphi(t)\phi_1(t) + \beta_1.$$

References

[1] Harris, T. E. The theory of branching processes. Englewood Cliffs, NJ: Prentice-Hall Applied Mathematics Series, 1963.

[2] Portier, C. J., & Sherman, C. K. A. Calculating tumor incidence rates in stochastic models of carcinogenesis. Math. Biosci., 135(2), 129–146(1996).
